# The University of London

**Published:** 1899-07-15

**Authors:** 


### THE UNIVERSITY OF LONDON.

THE senate of the University of London has given its assent to the proposals for establishing the University in the buildings of the Imperial Institute at South Kensington. As we understand the scheme, the whole of the Institute buildings are to be taken over by the Government, and in regard to the part to be assigned to the University the Government will stand directly in the relation of landlord to that body, the relation being the same as that which now exists in regard to the premises at present occupied by the University at Burlington House. The part of the Institute to be taken by the University includes the whole of the central building, and is said to place at its disposal for examinational and official work an area about five times as great as that provided at Burlington Gardens. What is wanted now is endowment.

